# Do the Sick Ever Sneeze?

**Published:** 1893-09

**Authors:** 


### Do the Sick Ever Sneeze?

"Do those who are seriously ill ever sneeze?" This is a point alluded to by Mr. Jonathan Hutchinson in the January number of his *Archives*. He does not recollect himself to have seen any but fairly healthy persons sneeze. He puts the question with especial reference to the widely spread popular superstition that sneezing is a sign of health and good luck. It is possible, he thinks, that this may have had its origin in the fact that it is for the most part an act restricted to those in fair health. Tylor, in his "Primitive Culture," gives interesting facts as to the prevalence of this creed and as to certain customs associated with it, and traces it in part to doctrines of animism, but Mr. Hutchinson thinks the suggestion he has given may also have some value.—*Sheffield Med. Journal.*

